# Supplementary figures and images for: A variant of Runx2 that differs from the bone isoform in its splicing is expressed in spermatogenic cells
Source: PeerJ. 2016 Apr 4;4:e1862. doi: 10.7717/peerj.1862 (PMC4824880; doi:10.7717/peerj.1862)

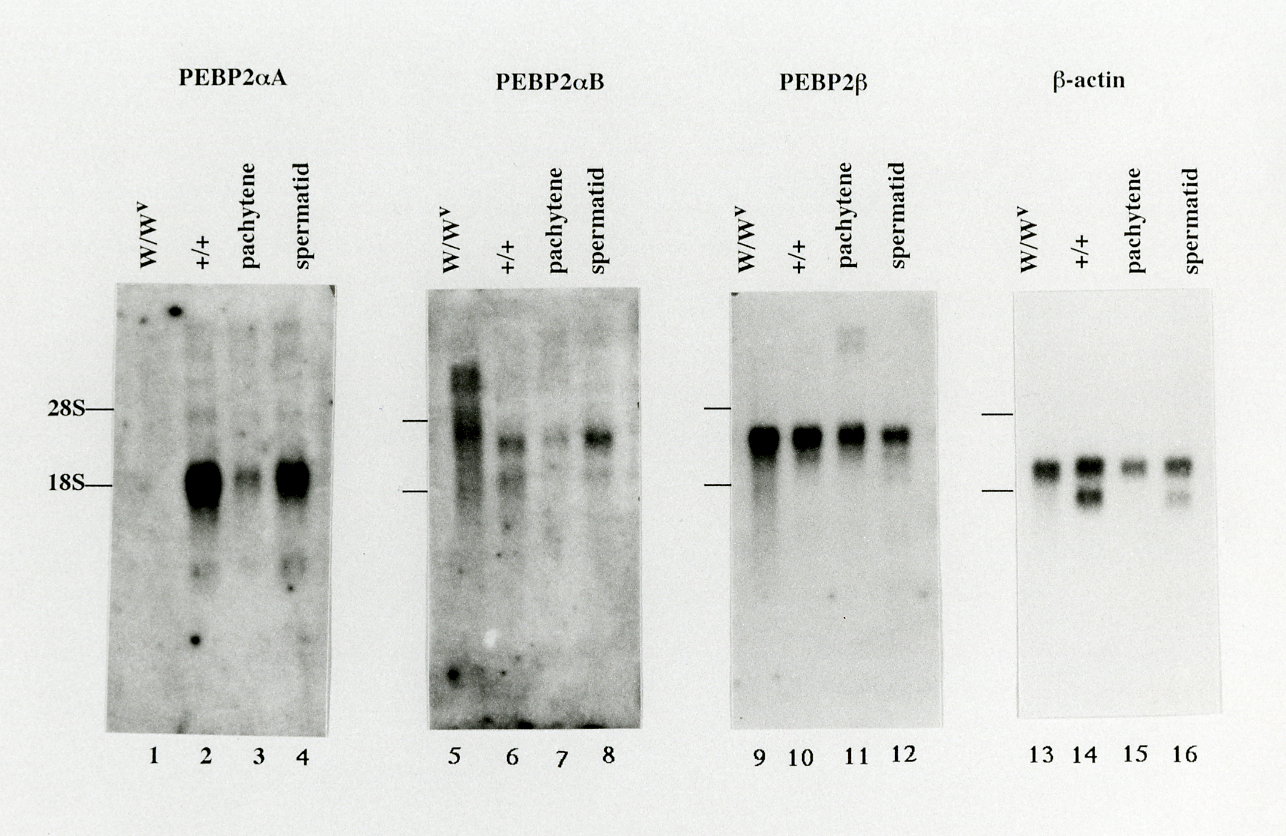

Supplement: Figure S1 — The Runx2 transcript was detected as a broad band of ∼1.8 kb length in wild-type testis, but not in W∕Wv testis (lanes 1 and 2). By contrast, Runx1 transcript (lanes 5 and 6) as well as PEBP2β/CBFβ transcript (lanes 9 and 10) and β-actin transcript used as loading control (lanes 13 and 14) were detected in both RNA samples tested. lane 1∼4: Runx2 (PEBP2αA), lane 5∼8: Runx1 (PEBP2αB), lane 9∼12: PEBP2 β/CBFβ, lane 13∼16: β-actin. [file peerj-04-1862-s001.png]

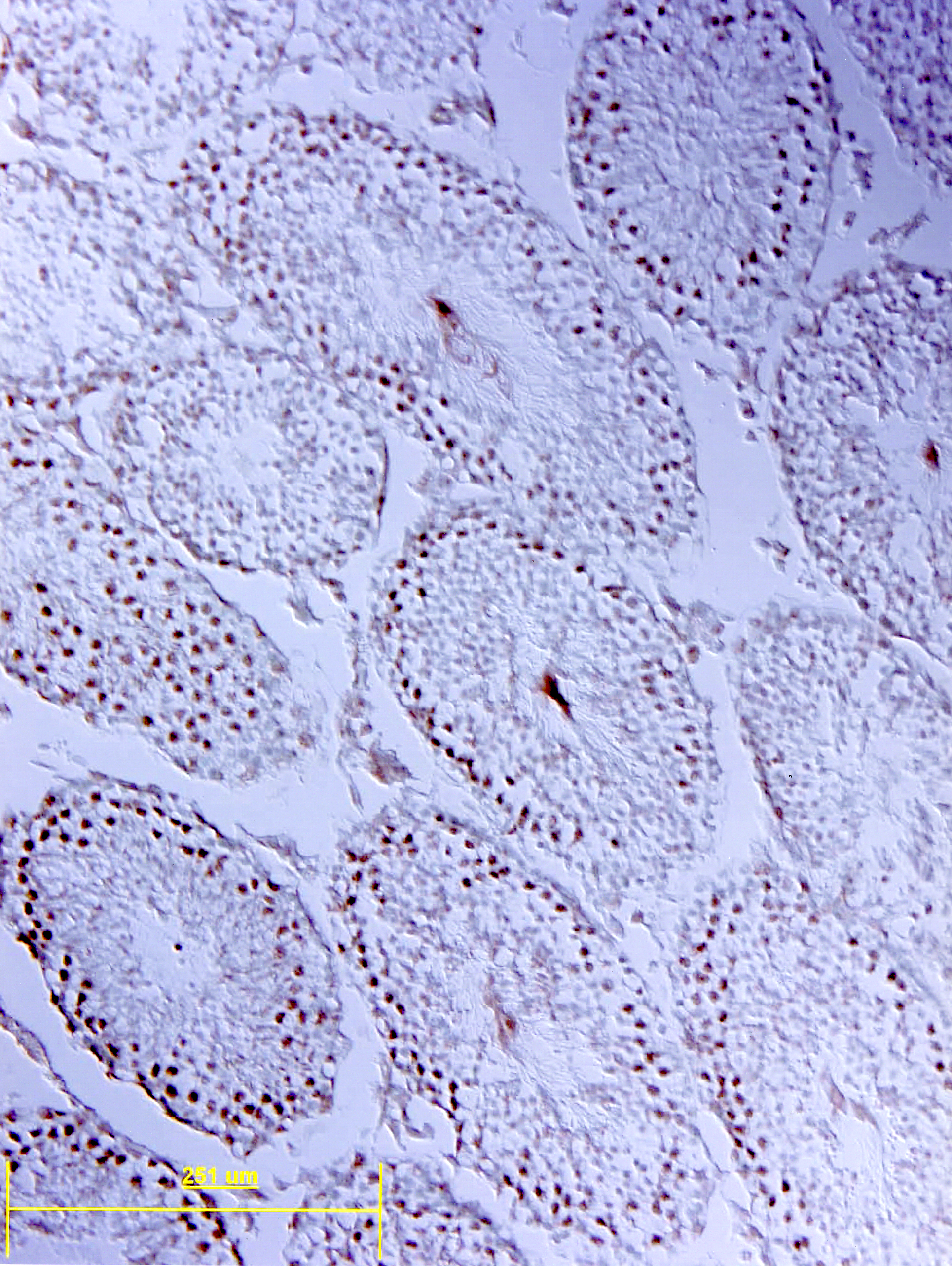

Supplement: Figure S2 — Immunohistochemical staining of Runx2 protein in testis is shown. Testes from adult C57BL/6J mice were stained with the anti-Runx2 antibody and counterstained with methyl green. [file peerj-04-1862-s002.png]

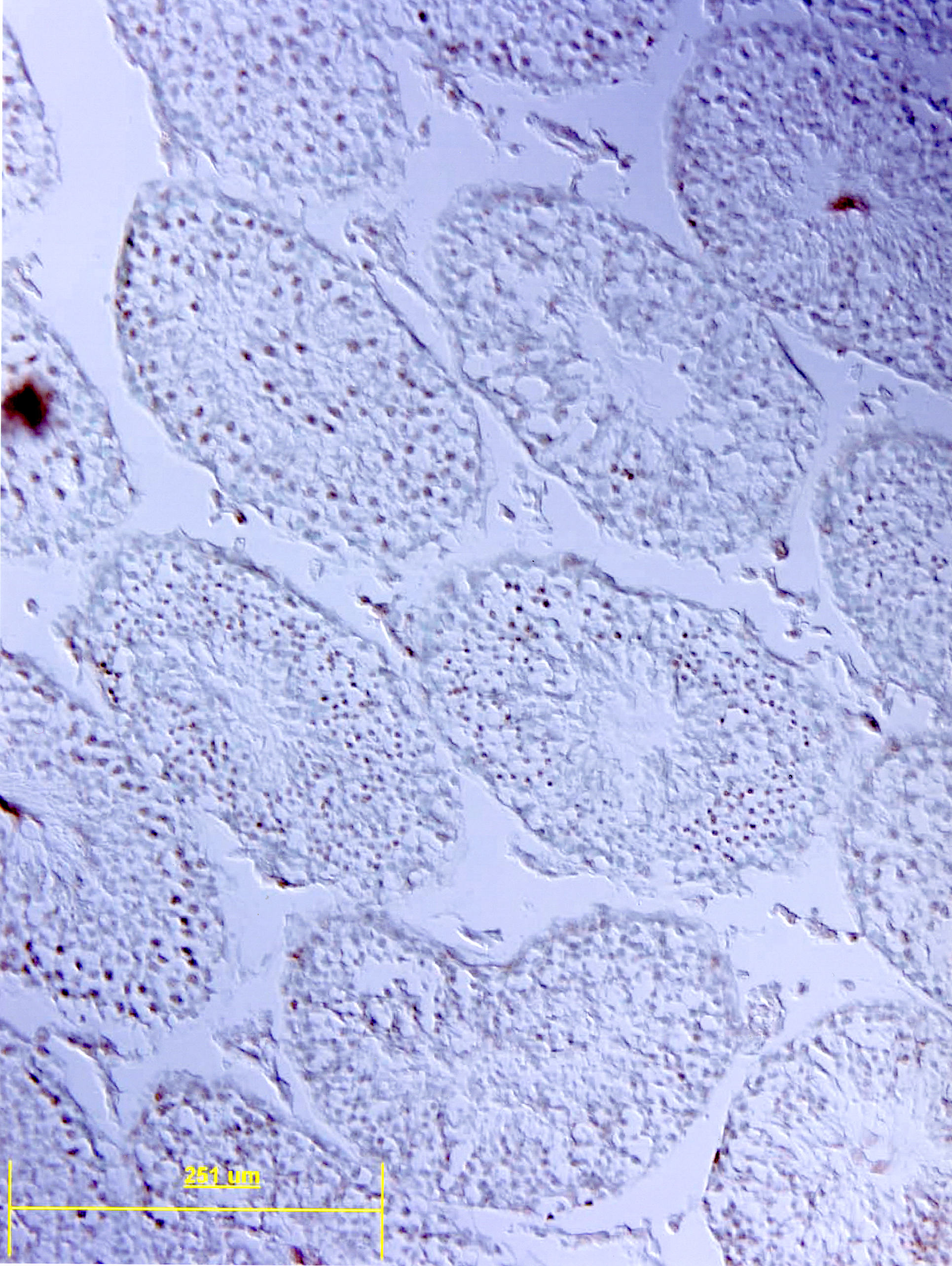

Supplement: Figure S3 — Immunohistochemical staining of Runx2 protein in testis is shown. Testes from adult C57BL/6J mice were stained with the anti-Runx2 antibody and counterstained with methyl green. [file peerj-04-1862-s003.png]
